# Comparison of aortic zones for endovascular bleeding control: age and sex differences

**DOI:** 10.1007/s00068-022-02033-7

**Published:** 2022-07-06

**Authors:** Boke Linso Sjirk Borger van der Burg, Suzanne Vrancken, Thijs Theodorus Cornelis Fransiscus van Dongen, Tom Wamsteker, Todd Rasmussen, Rigo Hoencamp

**Affiliations:** 1 Department of Surgery, Alrijne Hospital Leiderdorp, Simon Smitweg 1, 2353GA Leiderdorp, The Netherlands; 2 Defense Healthcare Organization, Ministry of Defense, Utrecht, The Netherlands; 3 Department of Surgery at Mayo Clinic, Rochester, USA; 4 Department of Surgery, Leiden University Medical Center, Leiden, The Netherlands; 5 Trauma Research Unit, Department of Surgery, Erasmus MC, University Medical Center Rotterdam, Rotterdam, The Netherlands

**Keywords:** REBOA, External landmark, Fluoroscopy free, Aortic zones

## Abstract

**Purpose:**

To gain insight into anatomical variations between sexes and different age groups in intraluminal distances and anatomical landmarks for correct insertion of resuscitative endovascular balloon occlusion of the aorta (REBOA) without fluoroscopic confirmation.

**Materials:**

All non-trauma patients receiving a computed tomography angiography (CT-A) scan of the aorta, iliac bifurcation and common femoral arteries from 2017 to 2019 were eligible for inclusion.

**Methods:**

Central luminal line distances from the common femoral artery (CFA) to the aortic occlusion zones were measured and diameters of mid zone I, II and III were registered. Anatomical landmarks and correlations were assessed. A simulated REBOA placement was performed using the Joint Trauma System Clinical Practice Guideline (JTSCPG).

**Results:**

In total, 250 patients were included. Central luminal line (CLL) measurements from mid CFA to aortic bifurcation (*p* = 0.000), CLL measurements from CFA to mid zone I, II and III (*p* = 0.000) and zone I length (*p* = 0.000) showed longer lengths in men. The length of zone I and III (*p* = 0.000), CLL distance measurements from the right CFA to mid zone I (*p* = 0.000) and II (*p* = 0.013) and aortic diameters measured at mid zone I, II and III increased in higher age groups (*p* = 0.000). Using the JTSCPG guideline, successful deployment occurred in 95/250 (38.0%) in zone III and 199/250 (79.6%) in zone I. Correlation between mid-sternum and zone I is 100%. Small volume aortic occlusion balloons (AOB) have poor occlusion rates in zone I (0–2.8%) and III (4.4–34.4%).

**Conclusions:**

Men and older age groups have longer CLL distances to zone I and III and introduction depths of AOB must be adjusted. The risk of not landing in zone III with standard introduction depths is high and balloon position for zone III REBOA is preferably confirmed using fluoroscopy. Mid-sternum can be used as a landmark in all patient groups for zone I. In older patients, balloon catheters with larger inflation volumes must be considered for aortic occlusion.

## Introduction

**Fig. 1:**
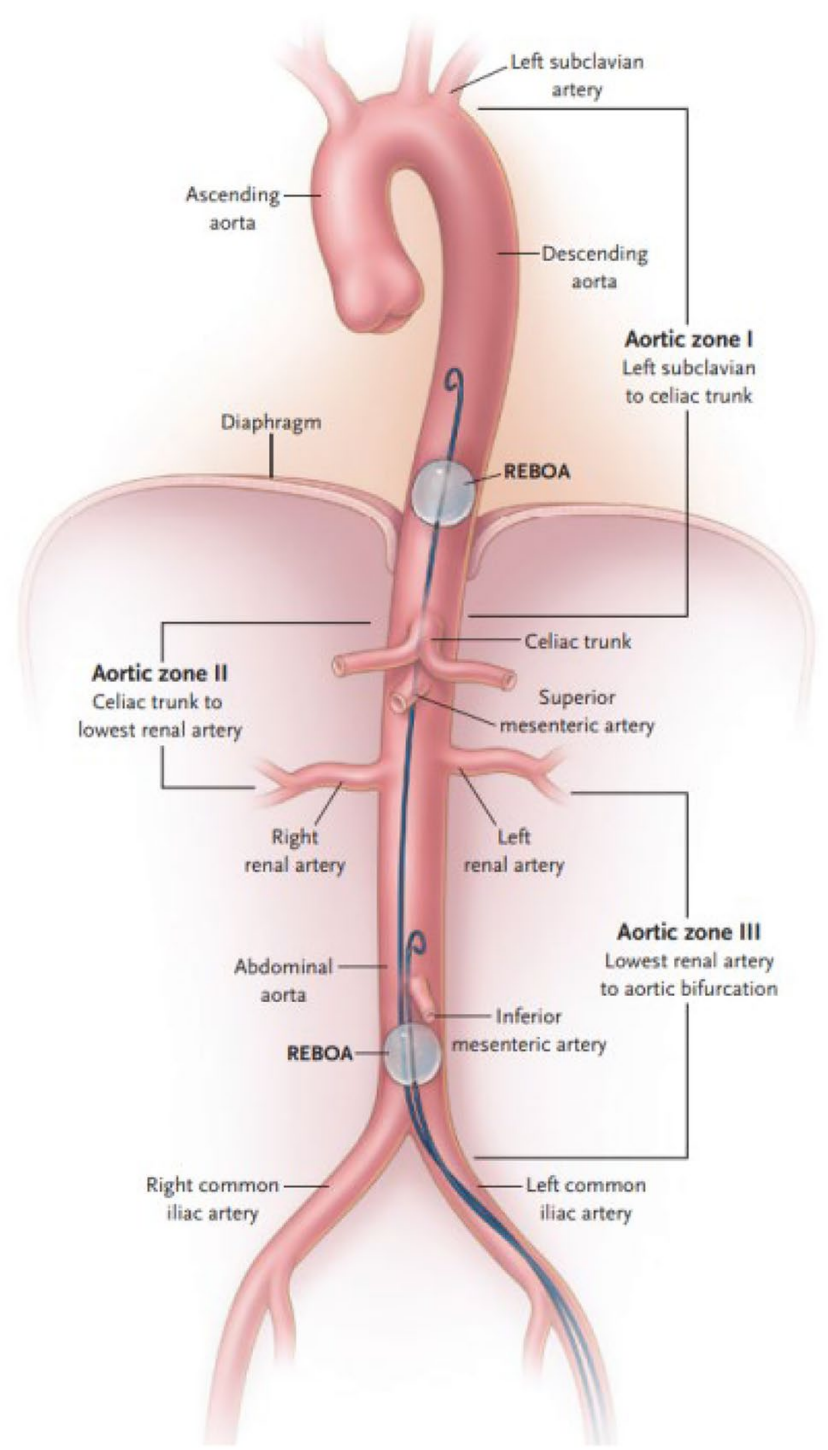
Aortic zones in the REBOA concept

Resuscitative endovascular balloon occlusion of the aorta (REBOA) is an adjunct for controlling non-compressible truncal hemorrhage (NCTH) [1]. The REBOA concept defines three aortic zones (Fig. 1) in which aortic occlusion balloons (AOB) can be placed [2]. REBOA can be performed with or without fluoroscopic confirmation of the desired location, with zone III for junctional and pelvic bleeding sources and zone I for sub-diaphragmatic injuries [3, 4]. Developments include the use of radiofrequency identification tags to confirm the placement [5]. The recently updated Joint Trauma System Clinical Practice Guideline (JTSCPG) and Eliason provided guidance for introduction depths to zone I and III to facilitate fluoroscopy-free introduction [6, 7]. Several studies present morphometric and CLL distances in relation to REBOA placement in young and predominantly male patients [7, 10].

The aim of this study was to gain insight into the variations of the aorto-iliac anatomy in a mixed male and female population of higher age to enhance safe REBOA insertion without fluoroscopic confirmation.

## Methods

**Fig. 2:**
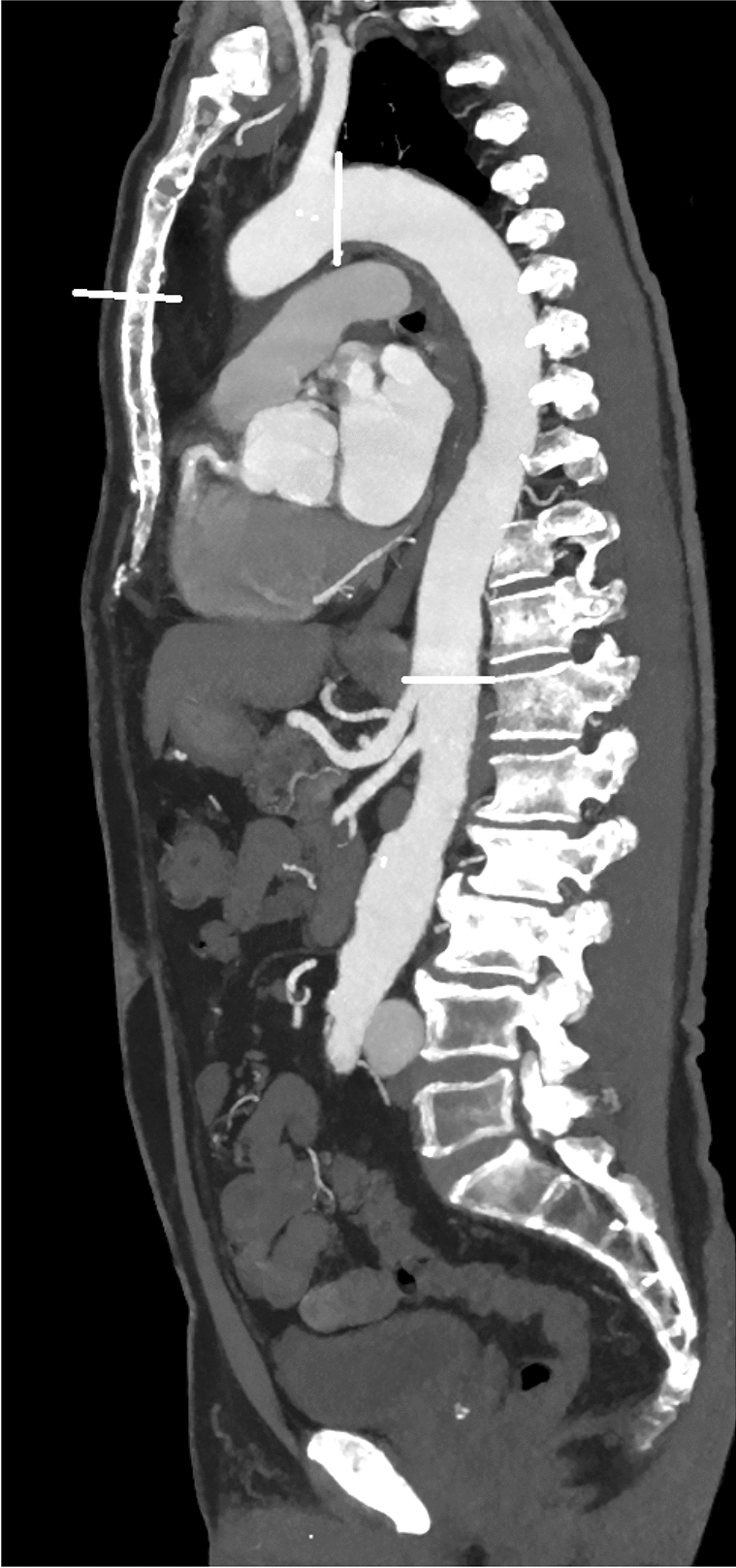
Mid-sternum projects to proximal, middle or distal third of zone I

This study was approved by the Medical Ethical Committee of Alrijne Hospital, the Netherlands (NWMO 18.114rt.tk). All patients undergoing a computed tomography angiography (CT-A) scan of the aorta, iliac bifurcation (AB) and common femoral arteries (CFA) from 2017 to 2019 were eligible for inclusion. Patients received a CT-A scan for various non-trauma indications. Age at CT-A scan, sex, presence and type of aortic disease and prior aortic surgery was registered. Using 3mensio Vascular^™^, Pie Medical Imaging, the Netherlands, a central luminal line (CLL) was constructed to determine intraluminal distances. The mid-section of the right CFA, indicating the arterial access site, was the starting point of measurements. Intraluminal distances from CFA to AB, from AB to renal arteries (RA) (zone III), from RA to celiac trunk (CT) (zone II) and from CT to left subclavian artery (LSA) (zone I) were recorded. Distances from mid CFA to mid zone I, II and III were measured. Aortic diameters of mid zone I, II and III were registered. Projection of mid-sternum to proximal, middle or distal third of zone I was assessed (Fig. 2), using Sectra Workstation IDS7, V3, Sectra AB, Sweden. A simulated REBOA placement in zone I and III was performed, applying introduction depths for zone I and III of the JTSCPG (46 and 28 cm) and Eliason (48 and 28 cm) [6, 7] to our cohort. Based on a previous study, the mean distance measured from skin to mid CFA, inclined according to the projected introducer sheath course, was 37 mm [7]. The length of the external part of a 7 fr sheath measured 20 mm. The ER-REBOA^™^ (Prytime Medical^™^ Devices, Inc) (40 mm balloon length) was used in this simulation. The middle of the balloon corresponds toh the introduction depth with 20 mm proximal and caudal extension.

Finally, total occlusion success rates of 17 commonly used aortic occlusion balloon (AOB) catheters [8] were determined for zone I and III.

### Statistical analysis

Statistical analyses were performed in collaboration with an expert (TD), using the Statistical Package for the Social Sciences (SPSS^®^, Version 24, IBM Corporation, Armonk, New York). Baseline information of the subjects was registered in an electronic file (Microsoft Excel^®^ and SPSS^®^). The *t* test (parametric) and Mann–Whitney *U* test (non-parametric) were used to analyze data. For all statistical analyses, a *p* value ≤ 0.05 was considered significant.

## Results

In total, 250 non-trauma patients were selected for inclusion from 2017 to 2019. Average age of included patients was 69.2 years (range 20–94 years) with 147/250 (58.8%) aged over 70 years old. We included 151 male patients (60.4%) and 99 female patients (39.6%). The presence of aneurysms, atherosclerosis, dissections or previous aortic interventions are listed in Table 1.

**Table 1:** Patient characteristics of 250 CT-A scans

Patient characteristics	Mean
Age in years (min–max)	69.2 (20–94 years)
Male (%)	151/250 (60.4%)
Female (%)	99/250 (39.6%)
Aneurysmatic aorta (%)	112/250 (44.8%)
Normal aorta (%)	107/250 (42.8%)
Atherosclerotic aorta (%)	20/250 (8%)
Dissected aorta (%)	11/250 (4.4%)
Post-intervention (%)	9/250 (3.6%)

### Zone lengths and central luminal line distances in relation to JTSCPG guidelines

CLL measurements from mid CFA to mid zone I, II and III showed significantly shorter lengths for women as compared to men; 251 mm (SD 23.3) for women and 274 mm (SD 28.0) for men from mid CFA to mid zone III and 462 mm (SD 36.0) for women and 497 mm (SD 39.3) in men from mid CFA to mid zone I (Table 2).

**Table 2:** Central luminal line measurements; lengths and diameters—*T* test

Central luminal line measurements	Male (*n* = 151) Mean in mm (SD)	Female (*n* = 99) Mean in mm (SD)	*p* value
Distance mid CFA–AB	222 (30.3)	202 (25.6)	0.000
Distance AB–distal origin of distal RA (length zone III)	103 (23.9)	97 (21.9)	ns
Distance distal RA–CT (length zone II)	42 (10.0)	42 (10.8)	ns
Distance of CT–distal origin of LSA (length zone I)	260 (30.0)	242 (31.8)	0.000
Distance mid CFA–mid zone III	274 (28.0)	251 (23.3)	0.000
Distance mid CFA–mid zone II	347 (30.8)	320 (26.3)	0.000
Distance mid CFA–mid zone I	497 (39.3)	462 (36.0)	0.000
Aorta diameter mid zone III	35 (20.9)	27 (17.4)	0.001
Aorta diameter mid zone II	26 (6.4)	23 (7.5)	0.002
Aorta diameter mid zone I	31 (7.6)	29 (9.6)	ns

*mm* millimeters, *SD* standard deviation, *CFA* common femoral artery, *AB* aortic bifurcation, *RA* renal artery, *CT* celiac trunc, *LSA* left subclavian artery, *ns* not significant

### JTSCPG guideline: introduction depths for zone III and I

Applying the JTSCPG guideline for zone III (28 cm) to our cohort, in 154/250 (62.4%) of patients (42.4% of women, 74.2% of men) zone III was not reached and in 1 female patient, 1/250 (0.4%), zone III had already ended. Applying the JTSCPG guideline for zone I (46 cm), 51/250 (20.4%) of patients (7.1% of women, 29.1% of men) did not reach zone I, resulting in (partial) deployment in zone II.

### Eliason’s adjustment of the JTSCPG guideline

The Eliason insertion lengths for zone III (28 cm) are identical to the JTSCPG guideline (Table 3). Applying Eliason’s 48 cm for zone I to our cohort, in 20/250 (8.0%) patients (2.0% of women and 11.9% of men) did not reach zone I, resulting in (partial) deployment in zone II.

**Table 3:** ABO introduction depths per zone; successful deployment in the desired zone

ABO introduction depths		Zone III (cm)	Successful deployment (n)	Zone I (cm)	Successful deployment (*n*)
JTSCPG	Female (*n* = 99)	27	39/99 (39.4%)	46	92/99 (92.9%)
	Male (*n* = 151)	27	21/151 (13.9%)	46	107/151 (70.9%)
	*Total* (*n* = 250)		60/250 (24.0%)		199/250 (79.6%)
Eliason	Female (*n* = 99)	28	56/99 (56.6%)	48	97/99 (98.0%)
	Male (*n* = 151)	28	39/151 (25.8%)	48	133/151 (88.1%)
	*Total* (*n* = 250)		95/250 (38.0%)		230/250 (92.0%)
Borger et al	Female (*n* = 99)	30.8	75/99 (75.8%)	51.9	97/99 (98.0%)
	Male (*n* = 151)	33.1	113/151 (74.8%)	55.4	150/151 (99.3%)
	*Total* (*n* = 250)		188/250 (75.2%)		247/250 (98.8%)

*ABO* aortic occlusion balloon, *cm* centimeters, *n* number

### Borger: introduction depths for zone III and I

We used our CLL length measurements from the right CFA to mid zone III and I for men and women separately for predicting successful mid zone III and I occlusion after a simulated introduction of an ER-REBOA^™^. In the female group, the average length from mid CFA to mid zone III was 251 mm. The AOB started at 288 mm (308 minus 20 mm) and extended to 328 mm (308 plus 20 mm). In women, the balloon had not reached the start of zone III in 13/99 (13.1%) and had already left zone III in 11/99 (11.1%) when introducing the AOB 251 mm. A balloon deployment of 308 mm deep reached zone III in 75/99 (75.8%) of our female population. The average length of zone III in men was 274 mm. The AOB started at 311 mm (331 minus 20 mm) and extended to 351 mm (331 plus 20 mm). In 19/151 (12.6%), the balloon did not reach zone III and in 19/151 (12.6%) zone III already ended. Therefore, a balloon deployment of 331 mm deep reached zone III in 113/151 (74.8%) of men. The average distance from mid CFA to mid zone I in women was 462 mm. No female patients projected caudally to zone I when a 40 mm balloon was deployed 519 mm deep. In 99/99 (100%), zone I was reached. In 2/99 (2.0%) zone I had already ended at 539 mm resulting in zone I placement in 97/99 (98%) of women. In two patients, the balloon projected 1 and 10 mm proximally to the distal origin of the LSA. This was not clinically relevant. In the male study group, the average mid zone I measured 497 mm. No male patients projected caudally to zone I when a 40 mm balloon was deployed 554 mm deep. In 1/151 (0.6%), zone I had already ended resulting in a 150/151 (99.3%) position in zone I. In this patient, balloon deployment of 554 mm deep projected 35 mm proximal to the LSA making it clinically relevant (Table 3).

When selecting the age group 20–50 years (mean 41 years) (*n* = 29) and using the JTSCPG guideline and Eliason insertion lengths, successful deployment in zone III occurred in 28/29 (96.6%). For JTSCPG zone I, successful deployment occurred in 28/29 (96.6%) and Eliason insertion lengths for zone I in 29/29 (100%).

### Variations and age

Dividing the cohort into three age groups (20–50, 51–70 and 71–94 years), differences in zone lengths, distances and diameters were determined. The length of zone I and III increased in elderly age groups. The length of zone I increased from a median of 237 mm [201–246; interquartile range] to 259 mm [242–273], *p* = 0.000. The length of zone III increased from a median of 88 mm [80–102] to 104 mm [91–118], *p* = 0.000. CLL distance measurements from the right CFA to mid zone I increased significantly (*p* = 0.013), from a median of 454 mm [435–479] to 487 mm [455–518]. CFA to mid zone III increased from a median of 251 mm [242–271] to 264 mm [245–286]. Aortic diameters measured at mid zone I increased significantly from 23 mm [21–26] to 30 mm [27–33] and diameters at mid zone III increased from 17 mm [14–19] to 37 mm [20–56] (Table 4).

**Table 4:** Central luminal line measurements per age group—MWU test

Age groups	20–50 years (*n* = 29)	51–70 years (*n* = 81)	71–94 years (*n* = 140)	*p* value^a^ (1–2)	*p* value^a^ (1–3)	*p* value^a^ (2–3)
Distance mid CFA–AB^b^	208 [197–222]	215 [198–233]	208 [191–232]	ns	ns	ns
Distance AB– distal origin of distal RA^b^ (length zone III)	88 [80–102]	96 [87–111]	104 [-91–118]	ns	0.000	0.049
Distance distal RA–CT (length zone II)	44 [36–51]	43 [38–47]	40 [34–46]	ns	ns	ns
Distance of CT–distal origin of LSA^b^ (length zone I)	237 [201–246]	246 [235–272]	259 [242–273]	0.000	0.000	0.041
Distance mid CFA–mid zone III^b^	251 [242–271]	263 [246–282]	264 [245–286]	ns	ns	ns
Distance mid CFA—mid zone II^b^	319 [302–338]	333 [317–354]	338 [312–363]	0.022	0.013	ns
Distance mid CFA—mid zone I^b^	454 [435–479]	480 [461–512]	487 [455–518]	0.001	0.000	ns
Aorta diameter mid zone 3^b^	17 [14–19]	22 [17–34]	37 [20–56]	0.000	0.000	0.000
Aorta diameter mid zone 2^b^	19 [17–23]	23 [21–34]	25 [22–28]	0.000	0.000	0.000
Aorta diameter mid zone 1^b^	23 [21–26]	27 [26–30]	30 [27–33]	0.000	0.000	0.000

Median distances in millimeters (IQR)

*MWU* Mann–Whitney *U* test, *IQR* interquartile range, *n* number, *CFA* common femoral artery, *AB* aortic bifurcation, *RA* renal artery, *CT* celiac trunc, *LSA* left subclavian artery, *ns* not significant

^a^Significant difference between age groups 1 (20–50), 2 (51–70) and 3 (71–94)

^b^Distances/diameters in mm

### Correlations

Anatomical measurements are listed in Table 5. Using mid-sternum as an external landmark, there is a 250/250 (100%) correlation between the projection of mid-sternum to zone I. There is no age effect.

**Table 5:** Anatomical measurements (*t* test) and correlations (MWU test) of 250 CT-A scans^a^

Correlation of mid-sternum to zone I	Male	Female	*P* value
Proximal 1/3 of zone I	94/151 (62.3%)	62/99 (62.6%)	ns
Middle 1/3 of zone I	57/151 (37.7%)	37/99 (37.4%)	ns
Distal 1/3 of zone I	0/151 (0%)	0/99 (0%)	ns

*MWU* Mann–Whitney U, *mm* millimeters, *SD* standard deviation, *PB* pubic bone, *XP* xyphoid process, *SSN* suprasternal notch, *LSA* left subclavian artery, *CFA* common femoral artery, *AB* aortic bifurcation, *ns* not significant

^a^In one female patient the SSN could not be identified on the CT-A scan

### Relation between aortic and balloon diameters

Calculated occlusion success rates are listed in Table 6. The REBOA Balloon^®^ 15 and REBOA Balloon^®^ 20 from REBOA Medical scored poorly with an occlusion rate below 3% in zone I and an occlusion rate of 11/250 (4.4%) versus 86/250 (34.4%) for zone III. The Q50® Plus Q50-65P, Medical and the Q50X^™^ Q50-65-X, QXMédical (Merit Medical) with 50 mm balloons had an occlusion rate of 199/250 (79.6%) for zone I and 239/250 (95.6%) for zone III.

**Table 6:** Balloon diameters of aortic occlusion balloon catheters for REBOA and occlusion rates in the study group

Aortic occlusion balloon	Max inflation *∅* (mm]	Occlusion success zone III (%)	Occlusion success zone I (%)
REBOA Balloon^®^ 15, REBOA Medical	15	11/250 (4.4%)	0/250 (0%)
REBOA Balloon^®^ 20, REBOA Medical	20	86/250 (34.4%)	7/250 (2.8%)
Equalizer^™^ Occlusion Balloon Catheter 17–105, Boston Scientific	20	86/250 (34.4%)	7/250 (2.8%)
Equalizer^™^ Occlusion Balloon Catheter 17–107, Boston Scientific	27	150/250 (60.0%)	85/250 (32.8%)
Fogarty^®^ Occlusion Catheter 62080814F, Edwards Lifesciences	28	154/250 (61.6)	107/250 (42.8%)
LeMaitre^®^ 2107–80 Aortic Occlusion Catheter, LeMaitre Vascular	28	154/250 (61.6)	107/250 (42.8%)
Coda^®^ 2–9.0–35-100–32, Cook Medical	32	163/250 (65.2%)	183/250 (73.2%)
ER-REBOA^™^, Prytime Medical Devices	32	163/250 (65.2%)	183/250 (73.2%)
Equalizer^™^ Occlusion Balloon Catheter 17–109, Boston Scientific	33	167/250 (66.8%)	198/250 (79.2%)
Equalizer^™^ Occlusion Balloon Catheter 17–111, Boston Scientific	40	183/250 (73.2%)	227/250 (90.8%)
Rescue balloon^™^ RB-167080-E, Tokai Medical Products	40	183/250 (73.2%)	227/250 (90.8%)
Fogarty^®^ Occlusion Catheter 62080822F, Edwards Lifesciences	45	191/250 (76.4%)	232/250 (92.8%)
LeMaitre^®^ 2107–81 Aortic Occlusion Catheter, LeMaitre Vascular	45	191/250 (76.4%)	232/250 (92.8%)
Coda^®^ 2–9.0–35-140–46, Cook Medical	46	195/250 (78.0%)	234/250 (93.6%)
Reliant^™^ Stent Graft Balloon Catheter, Medtronic	46	195/250 (78.0%)	234/250 (93.6%)
Q50^®^ Plus Q50-65P, QXMédical (Merit Medical)	50	199/250 (79.6%)	239/250 (95.6%)
Q50X^™^ Q50-65-X^a^, QXMédical (Merit Medical)	50	199/250 (79.6%)	239/250 (95.6%)

*AOB* aortic occlusion balloon, *mm* millimeter

^a^Currently only available in the USA

## Discussion

We report correlations between CLL distances, anatomical landmarks and aortic diameters for REBOA placement in a non-trauma population of higher age. This cohort describes patients with the highest average age compared to other cohorts [7, 10, 11]. CLL measurements from mid CFA zone I, II and III showed longer distances and lengths than other cohorts [7, 10, 11]. Older patients have longer zone lengths, longer distances to zone I and III and larger diameters in zone I and III. Correlation between mid-sternums to zone I is strong and can be used as an external anatomical landmark in these older patient groups. In older patients, larger aortic diameters can prevent occlusion with smaller AOBs.

CLL measurements from mid CFA to mid zone I, II and III showed significant longer lengths for men as compared to women. In our simulation, deployment of an ER-REBOA™ at the common segment for zone I and III predicted a higher successful aortic occlusion after introduction of an ER-REBOA^™^ in comparison to the JTSCPG and Eliason introduction depths that do not differentiate for age or sex. When only the age group 20–50 years (mean 41 years) was selected and using the JTSCPG and Eliason introduction depths, successful deployment occurred in 96.6% for zone III and in 96.6 and 100% for zone I, respectively. These findings confirm that our non-trauma population differs significantly from the cohorts on which the JTSCPG and Eliason guidelines are based and that AOB introduction depths should be adjusted for sex and age.

Several studies present morphometric and CLL distances in relation to REBOA placement in younger and predominantly male patients. None of these studies report on aneurysmatic patient groups or post-aortic surgery patients. Some studies present distance measurements from CFA to LSA (zone I) and CFA to renal artery (zone III) that include data from older patients [9, 11]. Similar to our data, the study of Kamenskiy et al. shows longer lengths in higher age groups. This study of vascular morphometry in patients from different age groups demonstrated an increase in arterial diameters and tortuosity with increasing age [11]. Another study showed that thoracic aorta length was significantly related to age and increased secondary to elongation by 59 mm (males) or 66 mm (females) between the ages of 20 and 80 years [12]. Olsen et al. identified potentially safe intervals for zone I REBOA with 99.7% likelihood between 43 and 48 cm in a series of 100 patients (mean age 78 years, 38% (*n* = 38) female patients) diagnosed with severe aortic stenosis planned for TAVI [13]. No similar potentially safe interval could be calculated for zone III. They did not provide the presence of aneurysms, atherosclerosis, dissections or previous interventions in their cohort. We identified introduction depths of 51.9 cm for females and 55.4 cm for men with 98.8% safety for placement of REBOA in zone I. This difference could be explained by a 44.8% presence of aneurysmatic aortas in our cohort. Correlations between mid-sternum and zone I were strong, with no patients projecting proximally or distally to aortic zone I. This confirms findings of a cadaver study that found a 100% likelihood using the mid-sternum as an external landmark for identification of zone I [14].

The AOBs with a diameter of 15 and 20 mm scored low occlusion rates in zone I and zone III. AOBs with the highest percentage of successful occlusion were with balloon diameters of 50 mm. The larger AOBs, including the Q50^®^ Plus Q50-65P, QXMédical (Merit Medical), are compliant balloons. In our cohort, aortic diameters were larger than other cohorts.

There are limitations to this study. Our cohort represents a non-trauma population of higher average age as compared to other cohorts. Older patients are at lower risk of sustaining NCTH-related injuries. There is a selection bias in our cohort because of the indication of the CT-A scan. Ideally, randomly included subjects from different age groups were compared.

## Conclusions

Men and older age groups have longer CLL distances to zone I and III. In older patients, AOB introduction depths must be adjusted. The risk of missing zone III in these patients is high and balloon position for zone III REBOA is preferably confirmed using fluoroscopy. Correlation between the projections of mid-sternum to zone I is very strong and can be used as an anatomical landmark in all patient groups in the absence of fluoroscopic confirmation. In these older patients, AOB with larger inflation volumes must be considered for successful occlusion of larger aortic diameters. Further research to determine ideal introduction depths for different age groups is warranted.
